# Repair Versus Replacement in Mitral Valve Papillary Muscle Rupture: A Multicenter Study

**DOI:** 10.1093/ejcts/ezaf284

**Published:** 2025-08-22

**Authors:** Giulio Massimi, Matteo Matteucci, Michele De Bonis, Mariusz Kowalewski, Francesco Formica, Claudio Francesco Russo, Sandro Sponga, Igor Vendramin, Andrea Colli, Giosuè Falcetta, Cinzia Trumello, Massimiliano Carrozzini, Theodor Fischlein, Giovanni Troise, Guglielmo Actis Dato, Stefano D’Alessandro, Peyman Sardari Nia, Vittoria Lodo, Emmanuel Villa, Shabir Hussain Shah, Roberto Scrofani, Irene Binaco, Jurij Matija Kalisnik, Marco Dell’Uomo, Matteo Pettinari, Matthias Thielmann, Bart Meyns, Fareed A Khouqeer, Carlo Fino, Caterina Simon, Andrea Musazzi, Adam Kowalowka, Marek A Deja, Angelo Pisani, Jakub Batko, Valentino Borghetti, Daniele Ronco, Michele Di Mauro, Roberto Lorusso

**Affiliations:** Department of Cardiothoracic Surgery, Heart and Vascular Centre, Maastricht University Medical Centre, Maastricht, 6221, The Netherlands; Cardiothoracic and Vascular Department, Santa Maria Hospital, Terni, Italy; Department of Cardiothoracic Surgery, Heart and Vascular Centre, Maastricht University Medical Centre, Maastricht, 6221, The Netherlands; Cardiac Surgery Unit, ASST Sette Laghi Circolo Hospital, Varese, Italy; Cardiothoracic Surgery Department, San Raffaele University Hospital, Milan, Italy; Department of Cardiothoracic Surgery, Heart and Vascular Centre, Maastricht University Medical Centre, Maastricht, 6221, The Netherlands; Thoracic Research Centre, Collegium Medicum, Nicolaus Copernicus University, Innovative Medical Forum, Bydgoszcz, Poland; Department of Medicine and Surgery, Cardiac Surgery Clinic, San Gerardo Hospital, University of Milano-Bicocca, Monza, Italy; Experimental Medicine Department, University of Salento, Lecce, Italy; Cardiac Surgery Unit, Cardio-Thoraco-Vascular Department, Niguarda Hospital, Milan, Italy; Cardiothoracic Department, University Hospital of Udine, Udine, Italy; Cardiothoracic Department, University Hospital of Udine, Udine, Italy; Section of Cardiac Surgery, University Hospital, Pisa, Italy; Section of Cardiac Surgery, University Hospital, Pisa, Italy; Cardiothoracic Surgery Department, San Raffaele University Hospital, Milan, Italy; Cardiac Surgery Unit, Cardio-Thoraco-Vascular Department, Niguarda Hospital, Milan, Italy; Department of Cardiac Surgery, Cardiovascular Center, Klinikum Nürnberg, Paracelsus Medical University, Nuremberg, Germany; Cardiac Surgery Unit, Poliambulanza Foundation Hospital, Brescia, Italy; Cardiac Surgery Department, Mauriziano Hospital, Turin, Italy; Department of Medicine and Surgery, Cardiac Surgery Clinic, San Gerardo Hospital, University of Milano-Bicocca, Monza, Italy; Department of Cardiothoracic Surgery, Heart and Vascular Centre, Maastricht University Medical Centre, Maastricht, 6221, The Netherlands; Cardiac Surgery Department, Mauriziano Hospital, Turin, Italy; Cardiac Surgery Unit, Poliambulanza Foundation Hospital, Brescia, Italy; Cardiovascular and Thoracic Surgery Department, King Fahad Medical City, Riyadh, Saudi Arabia; Cardiac Surgery Unit, Policlinico Milano Hospital, Milan, Italy; Cardiac Surgery Unit, Policlinico Milano Hospital, Milan, Italy; Department of Cardiac Surgery, Cardiovascular Center, Klinikum Nürnberg, Paracelsus Medical University, Nuremberg, Germany; Cardiothoracic and Vascular Department, Santa Maria Hospital, Terni, Italy; Department of Cardiovascular Surgery, Ziekenhuis Oost-Limburg, Genk, Belgium; Department of Thoracic and Cardiovascular Surgery, West-German Heart Center, University of Duisburg-Essen, Essen, Germany; Department of Cardiac Surgery, University Hospitals Leuven, Leuven, Belgium; Department of Cardiac Surgery, King Faisal Specialist Hospital and Research Center, Riyadh, Saudi Arabia; Cardiovascular Department, Papa Giovanni XXIII Hospital, Bergamo, Italy; Cardiovascular Department, Papa Giovanni XXIII Hospital, Bergamo, Italy; Cardiac Surgery Unit, ASST Sette Laghi Circolo Hospital, Varese, Italy; Department of Cardiac Surgery, Medical University of Silesia, Katowice, Poland; Department of Cardiac Surgery, Medical University of Silesia, Katowice, Poland; Department of Cardiac Surgery, Santa Maria della Misericordia Hospital, Perugia, Italy; Thoracic Research Centre, Collegium Medicum, Nicolaus Copernicus University, Innovative Medical Forum, Bydgoszcz, Poland; CAROL—Cardiothoracic Anatomy Research Operative Lab, Department of Cardiovascular Surgery and Transplantology, Institute of Cardiology, Jagiellonian University Medical College, Krakow, 31-007, Poland; Cardiothoracic and Vascular Department, Santa Maria Hospital, Terni, Italy; Department of Cardiothoracic Surgery, Heart and Vascular Centre, Maastricht University Medical Centre, Maastricht, 6221, The Netherlands; Cardiac Surgery Unit, Cardio-Thoraco-Vascular Department, Niguarda Hospital, Milan, Italy; Department of Cardiothoracic Surgery, Heart and Vascular Centre, Maastricht University Medical Centre, Maastricht, 6221, The Netherlands; Department of Cardiothoracic Surgery, Heart and Vascular Centre, Maastricht University Medical Centre, Maastricht, 6221, The Netherlands; Cardiovascular Research Institute Maastricht, Maastricht, 6221The Netherlands

**Keywords:** mitral valve, papillary muscle rupture, myocardial infarction, mitral valve replacement, mitral valve repair

## Abstract

**Objectives:**

Papillary muscle rupture (PMR) is a rare but potentially fatal mechanical complication after acute myocardial infarction (AMI). Although surgery is considered the gold-standard treatment for post-AMI PMR, the optimal surgical strategy remains unclear.

**Methods:**

Data from post-AMI PMR patients submitted to mitral valve replacement (MVR) or mitral valve repair (MVr) surgery in the period between 2001 and 2019, from 20 international centres, were collected in the CAUTION study database. In-hospital and long-term post-discharge mortality were the endpoints. A multivariable logistic regression model was used to determine mortality independent factors.

**Results:**

The patient cohort available included 218 patients. MVR was the most frequent type of surgery (81.6%). Complete PMR was more common in the MVR group (71.9%, *P* = .008), while partial PMR was more frequent in MVr patients (75%, *P* = .008). In-hospital mortality rate was 25.8% in the MVR subgroup and 20% in MVr subjects (*P* = .440). In MVR subgroup, concomitant coronary artery bypass grafting (CABG) was associated with lower in-hospital mortality (*n* = 20/96, 21%) than no concomitant CABG (31.7%, *P* = .035). Survival at 1, 3, 5, and 10 years was 59.3%, 55.9%, 53.1%, 46.9% in the MVR group and 59.9%, 56.8%, 54.1%, and 43.2% in MVr patients, respectively, with no statistical differences (*P* = .474). Patients underwent MVr surgery, and 1-, 3-, 5-, and 10-year survival was 79.8%, 75.4%, 68.5%, and 37.5%, respectively, when CABG revascularization was performed, while no CABG survival was 16.7%, 16.7%, 8.3%, and 8.3% (*P* < .001).

**Conclusions:**

MVR is the most commonly performed in complete post-AMI PMR and MVr in partial PMR. No differences were observed regarding in-hospital and long-term mortality in the 2 surgical groups, and no independent factors were associated with overall mortality. Concomitant CABG was associated with higher in-hospital survival.

**Clinical Registration Number:**

Clinicaltrials.gov, NCT03848429.

## Graphical Abstract

**Figure:**
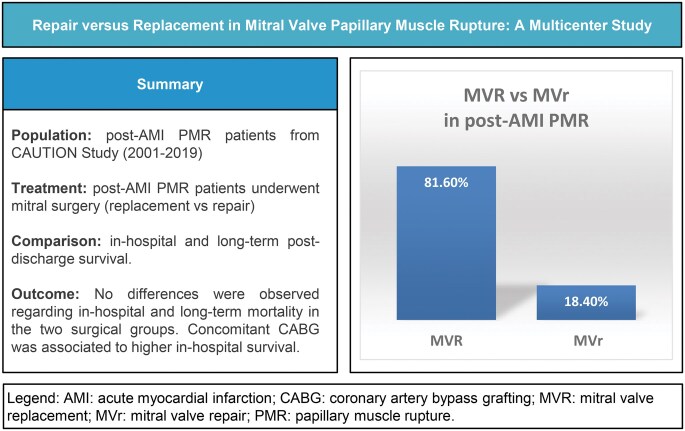

## INTRODUCTION

Papillary muscle rupture (PMR) is a rare but life-threatening complication of acute myocardial infarction (AMI). Compared with the myocardial pre-reperfusion era, recent studies have reported a constant reduction in the incidence of such a complication.^1^ The latest large reports on post-AMI mechanical complications describe a prevalence ranging from 0.05% to 0.26% of AMI patients.^1^ Clinical appearance usually develops within a week of the acute myocardial event.^1^^,^^2^ From a management standpoint, medical treatment of post-AMI PMR is characterized by an in-hospital mortality rate up to 80%. Surgery remains the gold standard in treatment for post-AMI PMR, although percutaneous transcatheter techniques are emerging as potential alternative options.^3^ Mitral valve replacement, either with a mechanical or a biological prosthesis implantation, remains the most prevalent procedure in post-AMI PMR.^3–7^ However, a few series have reported mitral valve repair (MVr) series in post-AMI PMR, showing technical feasibility and favourable results in selected patients with preserved left ventricular (LV) function and improved LV remodelling as well as overall outcome.^4–6^ The present study, therefore, aimed at analysing and comparing mitral valve replacement (MVR) versus MVr results in post-AMI PMR from an international muticentre study collecting almost 20 years of experience to provide more robust data about the 2 surgical strategies applied in this setting (Central Image).

## METHODS

### Study design and population

The study cohort consisted of patients retrieved from the database of the CAUTION study (“Mechanical Complications of Acute Myocardial Infarction: An International Multicentre Cohort Study”). The CAUTION study (trial registration: Clinicaltrials.gov, NCT03848429) is a retrospective, international, multicentre, observational trial aimed at evaluating the postoperative outcomes of patients surgically treated for post-AMI MCs. The patient population of this CAUTION sub-study consisted of the adult patients (>18 years old) who were surgically treated for post-AMI PMR, between January 2001 and December 2019 at 20 different centres worldwide (**Figure S1**). Detailed information about demographics, preoperative risk factors, operative details, postoperative hospital course, morbidity, and mortality of this patient cohort was analysed. The study was conducted in accordance with the guidelines of the Declaration of Helsinki, and the related protocol was authorized by the local ethical committee of the leading centre (Maastricht University Medical Centre METC 2018-0924) and thereafter by the ethical committees of each of the involved centres. The primary outcomes of this study were in-hospital and long-term mortality of the MVR and MVr groups, alone and compared with each other. In-hospital (or early) mortality was defined as all-cause death that occurred within 30 days from the intervention or during the hospitalization related to the operation, while late mortality >30 days or after hospital discharge. Intraoperative mortality was considered a death occurring during the surgical procedure. The secondary outcomes included the identification of prognostic factors for in-hospital mortality after MVR or MVr surgery for post-AMI PMR.

Rupture of the whole papillary muscle (body) was defined as ‘complete’ or ‘total’; a rupture involving one of the heads of the papillary muscle was considered ‘partial’. Cardiogenic shock was defined by systolic blood pressure <90 mmHg, mean arterial pressure <65 mmHg or requiring 2 or more vasopressor infusions for haemodynamic support. The extracorporeal life support (ECLS) implantation was considered when implanted preoperatively in case of haemodynamic instability at risk of evolution to cardiogenic shock or cardiac arrest. The present observational study was conducted following the STROBE (STrengthening the Reporting of Observational studies in Epidemiology) statement guidelines. According to each centre and to each country, follow-up data were collected either by access to regional or national healthcare registries or by direct phone call.

### Statistical analysis

The analysis was performed comparing early in-hospital mortality between MVr and MVR groups. Categorical variables were compared using the chi-squared test or Fisher’s exact test as appropriate, while continuous variables were compared using the Mann-Whitney *U*-test. An analysis of patterns was performed. Since a very limited number of secondary variables showed >10% (and anyway <30%) of missing values, analysis was carried out without multiple imputations for missing values. The selection of covariates to be included in the multivariable model was based on their clinical relevance. No collinearity was present in the final model. The final model was chosen based on the best discrimination power and calibration (**Figure S2**).

Additional analyses evaluated early in-hospital mortality stratified by whether concomitant coronary artery bypass grafting (CABG) was performed, whether mechanical circulatory support (MCS) was utilized (eg, Extracorporeal Membrane Oxygenation or Intra-Aortic Balloon Pump [IABP]). Covariate adjustments were made using multiple regression for continuous outcomes and logistic regression for binary outcomes. Data are presented as counts and percentages. ORs were calculated along with 95% CIs. A *P*-value of <.05 was considered statistically significant. All computations were performed using STATA MP version 13.0. Survival curves were constructed with the Kaplan-Meier method for both the hospital survivors only, with subgroup analyses, and were compared using the log-rank test.

## RESULTS

### Patients characteristics

Patients’ demographic and preoperative clinical characteristics are presented in **Table 1**. The median age was 68 years old in both MVR and MVr subgroups, with a prevalence of males in the MVR subgroup than the MVr one (74.4% vs 57.5%, *P* = .030). More than two-thirds of patients on admission had an electrocardiogram pattern of ST elevation myocardial infarction with a prevalent inferior AMI localization in both MVR and MVr subgroups (80.9% vs 70%, *P* = .128). At admission, more than 60% of post-AMI PMR patients presented haemodynamic instability, with a higher incidence in MVR than in MVr surgery (79.8% vs 62.5%, *P* = .021). Thus, about half of each subgroup was in preoperative cardiogenic shock (59% vs 45% in MVR and MVr surgeries, respectively), and cardiac arrest was 7.9% (*n* = 14) in the MVR subgroup and 15% (*n* = 6) in the MVr subgroup. Most patients received coronary angiography in both subgroups, and thrombolysis was performed more frequently in patients who underwent MVr instead of MVR (12.5% vs 1.7%, *P* = .005). Circulatory mechanical supports were almost requested in the MVR subgroup. Indeed, IABP and ECLS were, respectively, 65.7% (*n* = 117) and 5.6% (*n* = 10) in the MVR subgroup, while these data were 37.5% (*n* = 15, *P* = .001) and 0% in the MVr subgroup.

**Table 1.** Demographics and Preoperative Clinical Characteristics

	MVR (*n* = 178)	MVr (*n* = 40)	*P-*value
Median age (years)	70.0 (62.0-74.0)	69.0 (56.5-76.5)	1.000
Male (%)	133 (74.7)	23 (57.5)	**.030**
Hypertension (%)	113 (63.5)	23 (57.5)	.481
Diabetes mellitus (%)	41 (23)	7 (17.5)	.446
Dyslipidemia (%)	62 (34.8)	16 (40)	.538
Smoking (%)	79 (44.4)	14 (35)	.279
CKD (%)	36 (20.2)	8 (20)	.974
ECG pattern (%)			.128
STEMI	144 (80.9)	28 (70)	
nSTEMI	34 (19.1)	12 (30)	
AMI localization (%)			
Anterior	62 (34.8)	10 (25)	.223
Lateral	21 (11.8)	6 (15)	.579
Inferior	82 (46.1)	19 (47.5)	.870
Posterior	13 (7.3)	5 (12.5)	.284
Hemodynamics (%)			**.021**
Stable	36 (20.2)	15 (37.5)	
Unstable	142 (79.8)	25 (62.5)	
Cardiogenic shock (%)	105 (59)	18 (45)	.108
Cardiac arrest (%)	14 (7.9)	6 (15)	.162
Cardiac tamponade (%)	5 (2.8)	4 (10)	**.047**
Mean LVEF (%)	45.0 (35.0-53.0)	45.0 (35.0-60.0)	.634
Coronary angiography (%)	157 (88.2)	35 (87.5)	.901
PCI (%)	33 (18.5)	6 (15)	.598
Thrombolysis (%)	3 (1.7)	5 (12.5)	**.005**
IABP (%)	117 (65.7)	15 (37.5)	**.001**
ECLS (%)	10 (5.6)	0 (0)	.267
Rupture to surgery (h)	6.0 (2.8-24.0)	2.0 (1.0-20.5)	.981
Concomitant post-AMI MC	14 (7.9)	1 (2.5)	.242

Abbreviations: AMI = acute myocardial infarction; CDK = chronic kidney disease; ECG = electrocardiogram; ECLS = extracorporeal life support; IABP = intra-aortic balloon pump; LVEF = left ventricular ejection fraction; nSTEMI = non-ST elevation myocardial infarction; MC = mechanical complication; PCI = percutaneous coronary intervention; STEMI = ST elevation myocardial infarction.

### Operative and perioperative information

The post-AMI PMR patients who underwent MVR were 81.6% (*n* = 178), while patients who underwent MVr were 18.4% (*n* = 40). The incidence of complete PMR was higher in the MVR than in the MVr subgroup (71.9% vs 25%, *P* = .008), while partial PMR was higher in MVr patients (75% vs 28.1%, *P* = .008). Concomitant CABG was performed in 53.9% (*n* = 96) and 67.5% (*n* = 27), respectively, in the MVR and MVr subgroups (*P* = .119). Two cases of reintervention due to recurrence of PMR were reported in the MVr subgroup (*P* = .023). Low cardiac output syndrome (LCOS) was the most frequent complication in both subgroups (25.8%, *n* = 46 in MVR vs 15%, *n* = 6 in MVr). More data concerning operative and perioperative information are in **Table 2**.

**Table 2.** Operative and Perioperative Data

	MVR (*n* = 178)	MVr (*n* = 40)	*P*-value
Type of rupture (%)			
Partial PMR	50 (28.1)	30 (75)	**.008**
Complete PMR	128 (71.9)	10 (25)	
CPB time (min)	129.0 (105.0-162.0)	135.0 (98.0-182.0)	1.000
ACC time (min)	80.0 (62.0-103.3)	91.0 (66.0-116.5)	1.000
Concomitant CABG (%)	96 (53.9)	27 (67.5)	.119
Postoperative inotropes (%)	123 (69.1)	23 (57.5)	.160
Postoperative IABP (%)	39 (21.9)	6 (15)	.331
Post-cardiotomy ECLS (%)	10 (5.6)	3 (7.5)	.650
Rethoracotomy for bleeding* (%)	17 (9.5)	3 (7.5)	.685
Reoperation* (%)	12 (6.7)	2 (5)	.685
Re-rupture*			
Recurrence	—	2 (5)	**.023**
Reintervention required	—	2 (5)	**.023**
ICU stay (days)	5.8 (2.0-14.0)	4.0 (3.0-9.0)	1.000
CVVHDF (%)	39 (21.9)	5 (12.5)	.184
LCOS (%)	46 (25.8)	6 (15)	.149
Postoperative cerebral event (%)	15 (8.4)	3 (7.5)	.847
Postoperative LVEF (%)	45.0 (40.0-54.0)	48.0 (35.0-54.5)	1.000

Abbreviations: ACC = aortic cross-clamp; CABG = coronary artery bypass; CPB = cardiopulmonary bypass; CVVHDF = continuous venovenous hemodiafiltration; ECLS = extracorporeal life support; IABP = intra-aortic balloon pump; ICU = intensive care unit; LCOS = low cardiac output syndrome; LVEF = left ventricular ejection fraction; PMR = papillary muscle rupture.

### In-hospital outcomes

**Figure 1.**
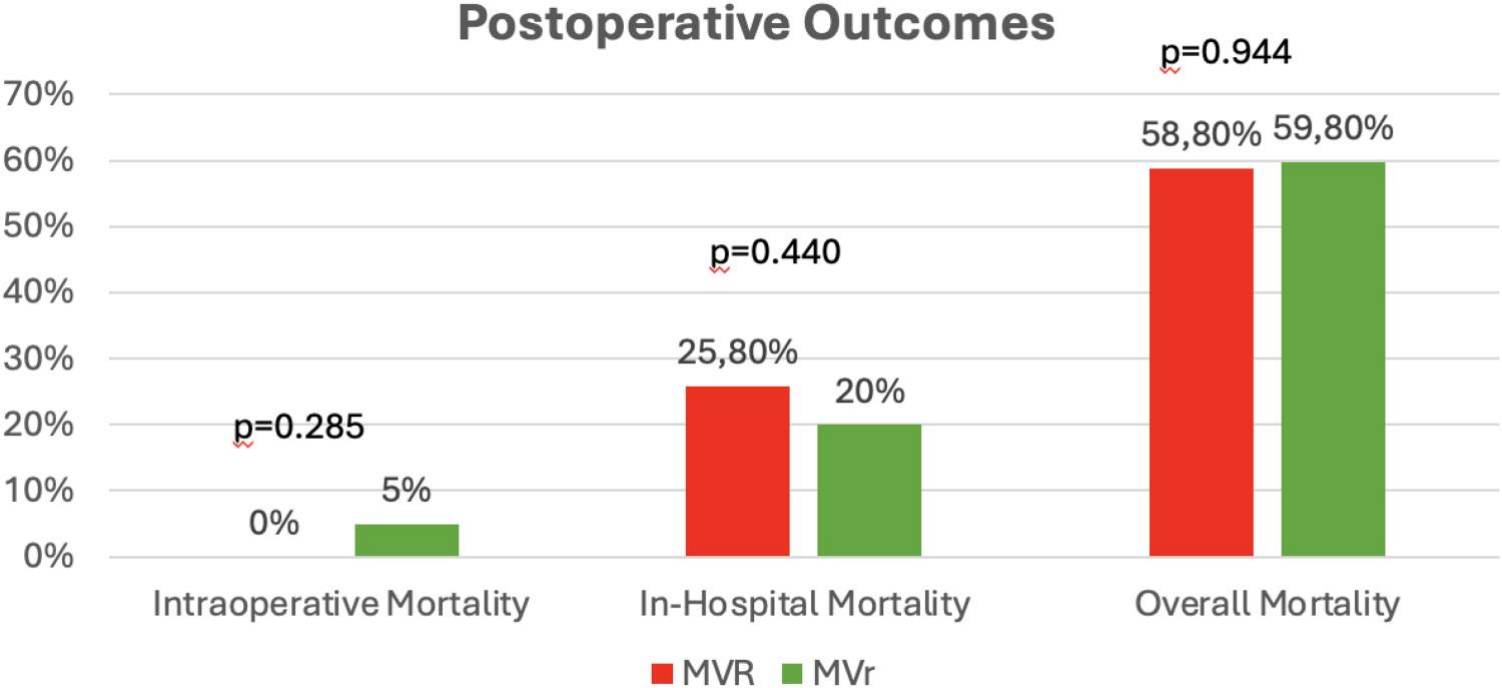
Postoperative Outcomes in MVR and MVr in Post-AMI PMR Populations. Abbreviations: AMI = acute myocardial infarction; MVR = mitral valve replacement; MVr = mitral valve repair; PMR = papillary muscle rupture

Intraoperative mortality was recorded only in 9 patients (5%), all underwent MVR, no cases were reported in the MVr group (*P* = .285; **Figure 1**). In-hospital mortality rate was 25.8% (*n* = 46/178) in the MVR subgroup and 20% (*n* = 8/40) in the MVr one, without statistical differences (*P* = .440; **Figure 1**). As causes of death, LCOS and multiorgan failure were more frequent after MVR than after MVr, without a statistical difference (**Table 3**). In the MVR subgroup, concomitant CABG was associated with lower in-hospital mortality (21%) than no concomitant CABG (31.7%) (*n* = 20/96 vs *n* = 26/82; OD: 0.48; CI, 0.25-0.95; *P* = .035). Also, in the MVr subgroup, concomitant CABG was associated with even lower in-hospital mortality (15%) (*n* = 4/27), if compared to the no CABG MVr subgroup, which was 30.8% (*n* = 4/13), but not significant (OD: 0.39; CI, 0.08-1.91; *P* = .246). Application of MCS (linked IABP and ECLS patients) was related to a lower in-hospital mortality in both groups, but without statistical differences. Indeed, in-hospital mortality related to concomitant MCS in MVR was 25.6% (*n* = 42/164) versus 28.6% in MVR without MCS (*n* = 4/14, *P* = .296), while in-hospital mortality in MVr group with MCS was 10% (*n* = 2/22) versus 30% in MVr without MCS (*n* = 6/18, *P* = .206).

**Table 3.** Outcome and Cause of Death

	MVR (*n* = 178)	MVr (*n* = 40)	*P*-value
Intraoperative mortality	9 (5)	0 (0)	.285
In-hospital mortality	46 (25.8)	8 (20)	.440
Discharge POD	15.0 (10.0-30.0)	10.0 (7.0-15.0)	1.000
Causes of death (%)			
Haemorrhagic shock	1 (2.2)	0 (0)	.817
CPB unweaning	2 (4.3)	0 (0)	.930
LCOS	13 (28.3)	2 (5)	.604
MOF	9 (19.6)	1 (2.5)	.491
CVA	2 (4.3)	0 (0)	.930
Sepsis	3 (6.5)	2 (5)	.219
AMI	2 (4.3)	0 (0)	.930
AKI	3 (6.5)	0 (0)	.752
Pneumonia	2 (4.3)	2 (5)	.119
Other	1 (2.2)	1 (2.5)	.273
Unknown	8 (15.3)	0 (0)	.327
Late mortality^a^	33 (24.3)	14 (37.8)	.101
Overall mortality^a^	80 (58.8)	22 (59.5)	.944

Abbreviations: AMI = acute myocardial infarction; AKI = acute kidney injury; CBP = cardiopulmonary bypass; CVA = cardiovascular accident; LCOS = low cardiac output syndrome; MOF = multiorgan failure; POD = postoperative days.

a On a total of 136 patients in MVR and 37 patients in MVr at follow-up.

### Long-term survival

**Figure 2.**
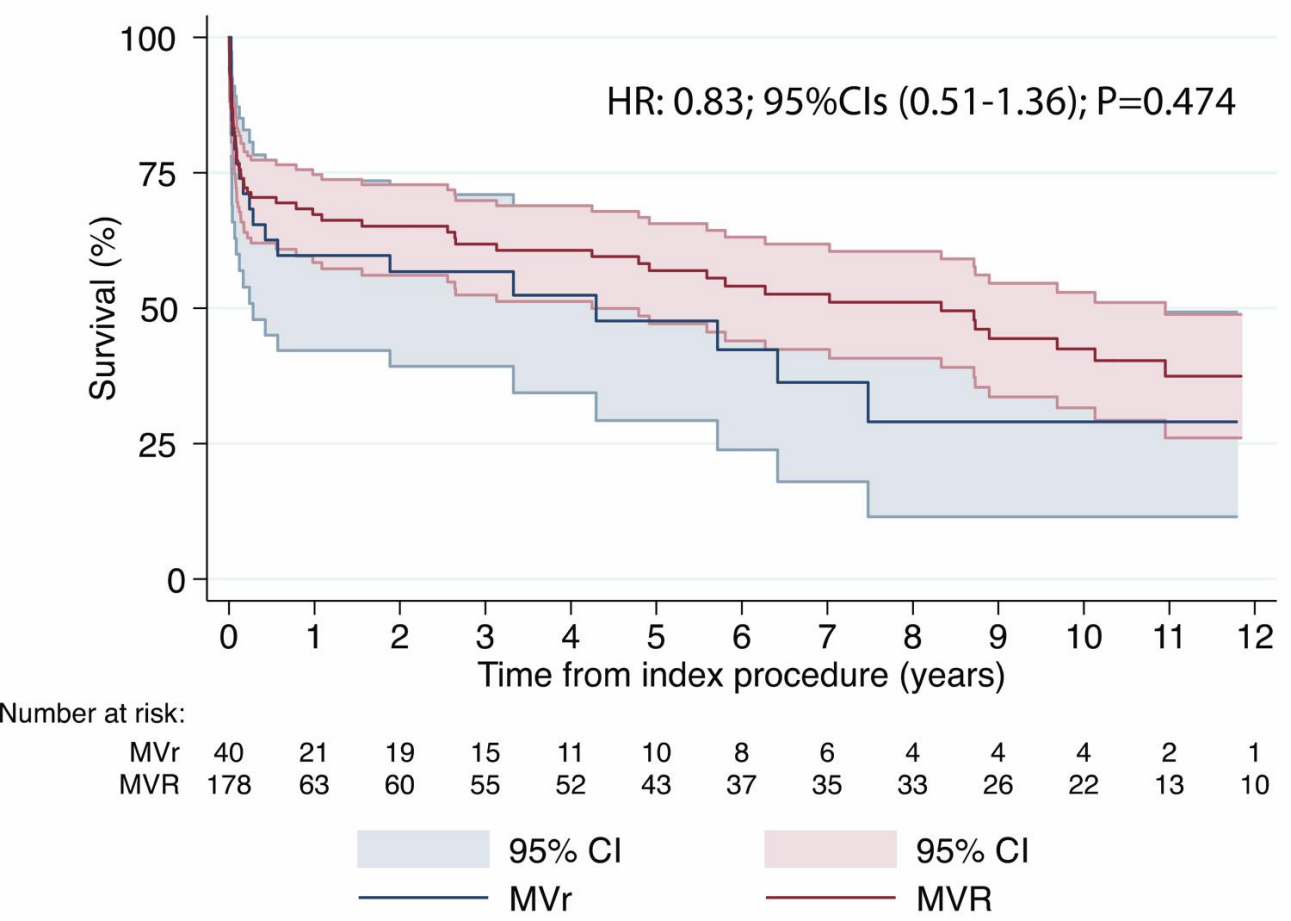
Kaplan-Meier Curves of Survival for MVR and MVr in Post-AMI PMR Population. Abbreviations: AMI = acute myocardial infarction; MVR = mitral valve replacement; MVr = mitral valve repair; PMR = papillary muscle rupture

**Figure 3.**
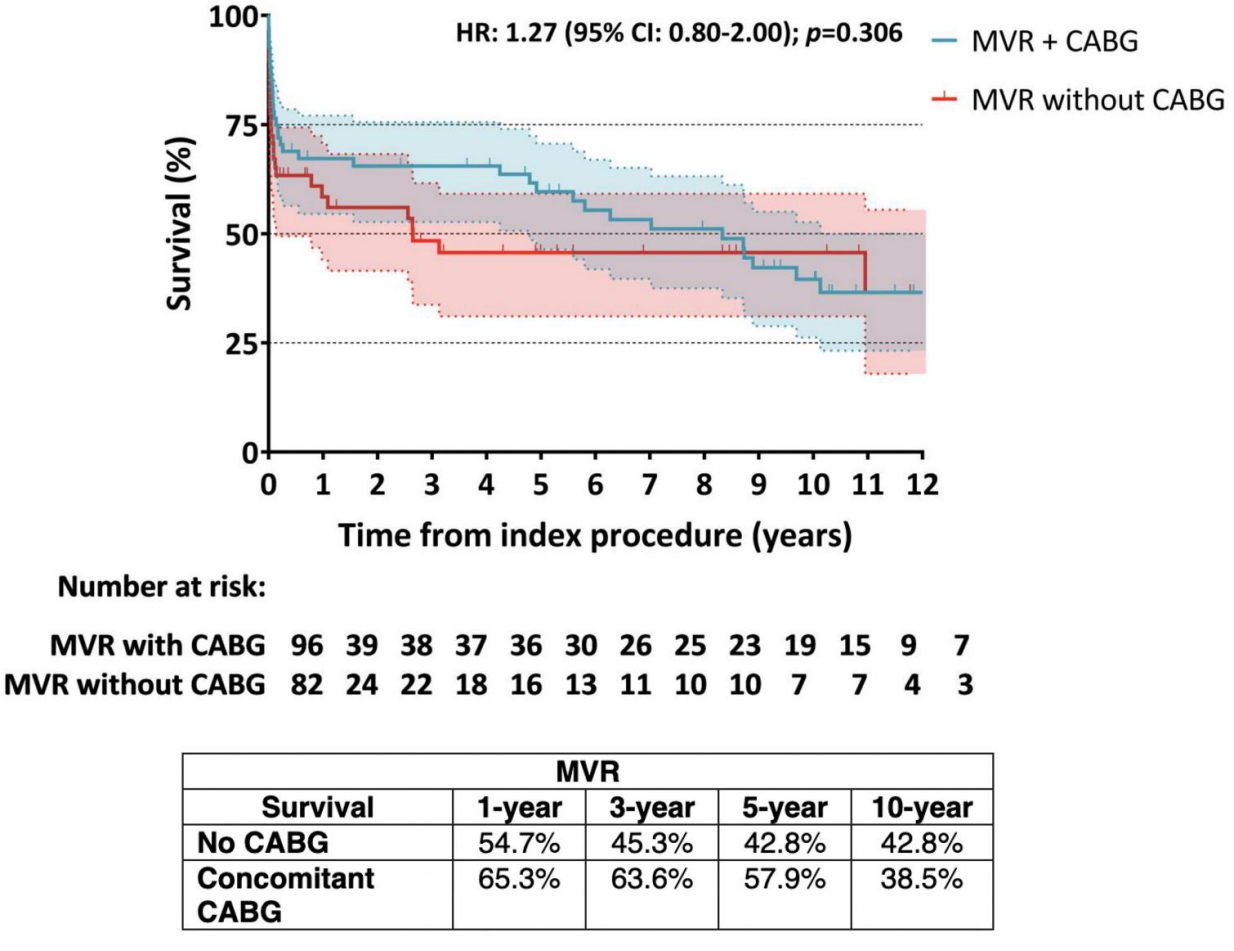
Kaplan-Meier Curves of Survival for Post-AMI Patients Who Underwent MVR With and Without Concomitant CABG Revascularization. AMI = acute myocardial infarction; CABG = coronary artery bypass grafting; MVR = mitral valve replacement

**Figure 4.**
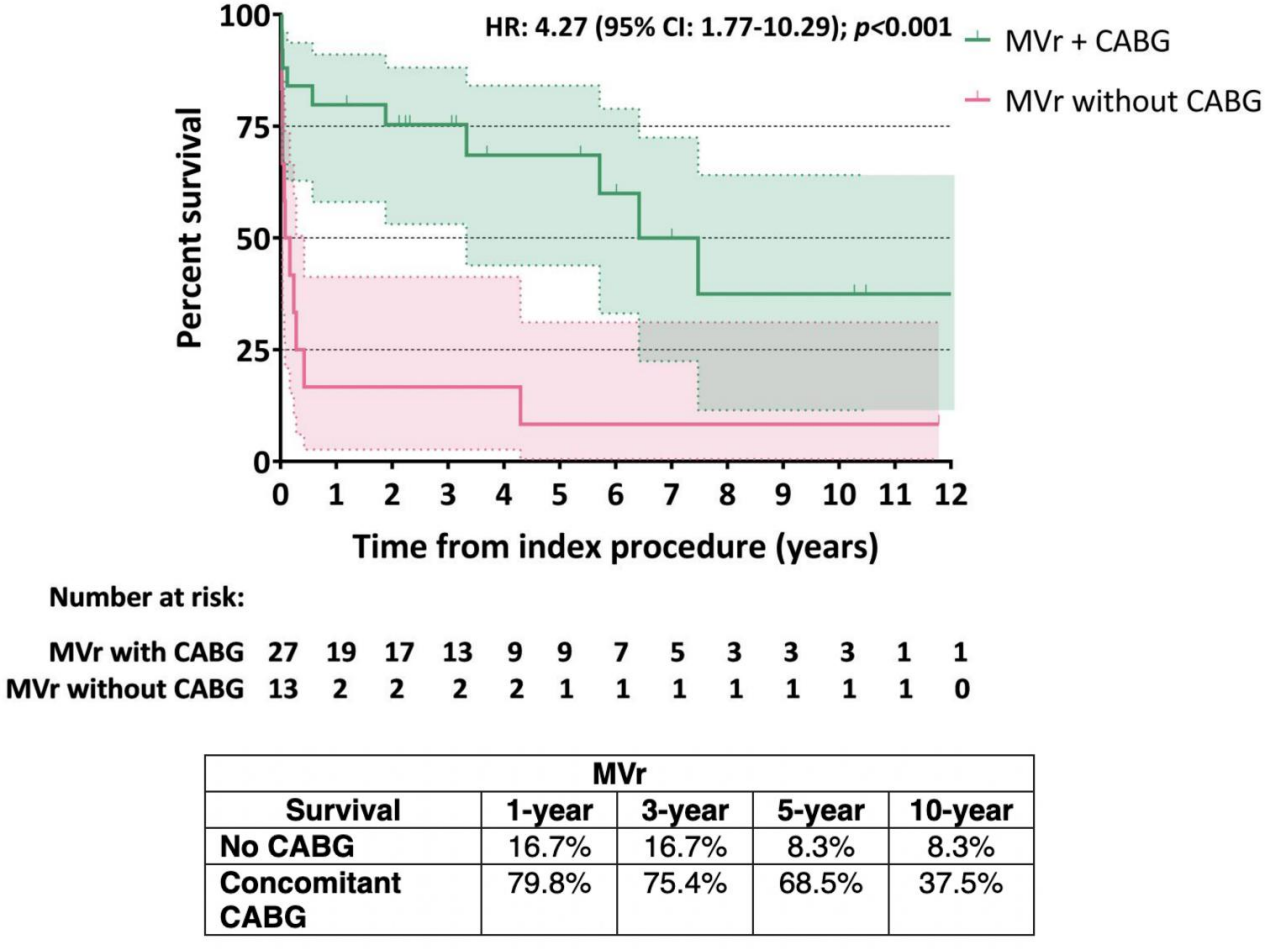
Kaplan-Meier Curves of Survival for Post-AMI Patients Who Underwent MVr With and Without Concomitant CABG Revascularization. Abbreviations: AMI = acute myocardial infarction; CABG = coronary artery bypass grafting; MVr = mitral valve repair

The mean follow-up time was 3 ± 4 years. Late mortality was 24.3% (*n* = 33/136) in the MVR group, while in the MVr one was 37.8% (*n* = 14/37), without statistical differences (*P* = .101; **Figure 1**). Concerning overall mortality, it was similar between the 2 groups (58,8%, *n* = 80/178 in the MVR group vs 59.5%, *n* = 22/40 in the MVr group, *P* = .944; **Figure 1**). For the 2 groups, survival at 1, 3, 5, and 10 years (**Figure 2**) was 59.3%, 55.9%, 53.1%, and 46.9% in the MVR group and 59.9%, 56.8%, 54.1%, and 43.2% in the MVr one, respectively, without statistical differences (OR: 0.83; CI, 0.51-1.36; *P* = .474). Regarding concomitant CABG, the 1-, 3-, 5-, and 10-year survival in the MVR group was 65.3%, 63.6%, 57.9%, and 38.5%, respectively, if concomitant CABG was performed and 54.7%, 45.3%, 42.8%, and 42.8% when CABG was not performed (**Figure 3**). Instead, patients underwent MVr surgery, and 1-, 3-, 5-, and 10-year survival was 79.8%, 75.4%, 68.5%, and 37.5%, respectively, when CABG revascularization was performed, while no CABG survival was 16.7%, 16.7%, 8.3%, and 8.3% (**Figure 4**).

### Predictors of mortality

Univariate and multivariate logistic regression analyses of in-hospital mortality predictors of post-AMI PMR MVR and MVr groups are shown in **Table S1**. Age and dyslipidaemia were significant at univariate analysis, without reaching statistical significance criteria at multivariate analysis.

## DISCUSSION

Post-AMI PMR patients who underwent surgical procedures still represent a challenge with rather high perioperative mortality.^7–10^ Valve replacement or repair represents possible approaches in this setting; however, comprehensive information about the outcomes of these different surgical strategies is reported in only a few publications and with limited patient cohorts.^4^^,^^6^^,^^9^^,^^10^ To the best of our knowledge, this is the first multicentre, retrospective, international study which addressed a more thorough analysis of the in-hospital and late outcomes of the 2 main surgical approaches in the presence of post-AMI PMR. The main findings of our study may be summarized as follows: (1) male patients, preoperative haemodynamic instability and preoperative IABP implant was associated more frequently with post-AMI PMR patients underwent MVR surgery; (2) cardiac tamponade and preoperative thrombolysis was associated with post-AMI PMR patients underwent MVr surgical procedure; (3) MVr surgery was performed more frequently in case of partial post-AMI PMR, while complete PMR is usually chosen with MVR surgery; (4) PMR recurrence was higher after MVr than MVR surgery; and (5) concomitant CABG surgery in post-AMI PMR patients improves in-hospital and long-term mortality with a statistical difference in MVr surgery. Literature suggests that most PMR patients undergo MVR, differently from what is suggested for patients with MR due to other causes.^6^^,^^7^^,^^9–12^ Nevertheless, recent reports have described a slightly increasing trend towards MVr also in this very peculiar and challenging situation. Although it is possible that the groups of patients receiving either procedure are different from each other in various aspects, the indications for PMR treatment are so limited that it is also possible that most patients undergo straightforward MVR, independent of their baseline characteristics. Moreover, some previous reports have also suggested a potentially more favourable outcome with MVR compared to MVr for certain high-risk patients with ischaemic cardiomyopathy and “ventricular” mitral regurgitation.^3–7^ Therefore, to better investigate the peculiar features of either group, we have analysed the PMR population of the CAUTION study also comparing the MVR vs MVr cohorts, to identify potentially relevant characteristics that might anticipate the feasibility of MVr or that might suggest areas of improvement in the surgical management of this condition.

### Mitral valve replacement in post-AMI PMR

In our study, we point out that there was a significantly higher incidence of male patients who underwent MVR, while the other preoperative characteristics were in accord with Pala et al.^6^ MVR is usually reserved for patients with complete PMR or partial PMR with fragile and/or extended infarcted tissue. MVR is also chosen in case of a patient’s preoperative critical clinical condition^6^^,^^7^^,^^9^^,^^10^^,^^13^ to reduce surgical procedure time and their related risks of complication. According to this, in our study, MVR was performed more frequently in haemodynamic instability if compared to MVr. In this way, and according to Pala et al,^6^ application of MCS as a bridge to surgery as preoperative IABP implant was significantly higher in patients who underwent MVR. Furthermore, preoperative ECLS was exclusively reported in MVR patients in our study. Surprisingly, preoperative cardiac tamponade was higher in patients who underwent MVr, but the different possible causes were not included in our study dataset, so further studies are needed.

### MVr in post-AMI PMR

Several publications reported in the literature advocate MVr in post-AMI PMR selected patients.^3^^,^^4^^,^^5^^,^^7^ A careful assessment and understanding of post-AMI PMR type and procedural concerns about necrosis extension of left ventricle wall are mandatory to avoid possible negative MVr results. As reported in the literature, partial PMR is usually associated with MVr surgery in our analysis.^4–7^ Only selected mitral valve insufficiency with complete PMR could be repaired. It could be feasible when the infarcted area is extremely localized and allows a safe PM reimplantation as an issue on its corresponding site, on the left ventricle wall or in the corresponding ‘healthy’ PM.^3^^,^^12^^,^^14^ In our opinion, to reduce the extension of the infarcted area, timely diagnosis and preoperative revascularization might play a role in patient suitability for MVr. According to this, we found a higher association of thrombolysis (statistically significant) and percutaneous coronary intervention with MVr than MVR surgery. Risk of a recurrence of PMR (re-PMR) after surgery is described not only after an MVr but also rarely after MVR surgery, when the leaflet preservation of the subvalvular apparatus is performed, due to the possibility of traction and tearing at the remainder PM. Anyway, the preservation of subvalvular apparatus has become a standard procedure in MVR to support the postoperative LV contractile function and improve outcome.^3^^,^^6^^,^^13^^,^^15^ Our study confirmed a low rate of re-PMR at all. We report a re-PMR in 5% of patients who underwent MVr and no cases after MVR surgery.

### Concomitant surgical revascularization

Concomitant CABG has no established consensus.^7^^,^^11^^,^^12^^,^^16^ The reasons are different. Sometimes, due to a critical condition, post-AMI PMR patients are led directly to the operating room without a coronary angiogram being performed. Other times, in post-AMI PMR patients with single-branch lesions and not extensive infarcted area, surgical revascularization is not performed because considered futile (especially if coronary disease is related to an infarcted area), to reduce operating time and its related risk. In this way, recent meta-analysis of PMR patients reported that concomitant CABG did not influence in-hospital survival,^11^ while a previous analysis on the overall cohort of CAUTION PMR patients indicated concomitant CABG as an independent predictor of early survival.^7^ Our data show a significant statistical difference in the MVr group between concomitant CABG and no CABG subgroups, with a better in-hospital outcome when post-AMI PMR patients were surgically revascularized. It could refute the concept of the futility of revascularization single-branch disease or small infarcted area (which are usually associated with MVr surgery patients’ suitability). Also, revascularization in MVR surgeries was associated with a lower in-hospital mortality, without reaching a statistical significance. Based on this assumption, we believe that concomitant CABG should be performed, when feasible, to improve survival.

### Mechanical circulatory supports

MCS has been proposed to improve extremely poor preoperative and intra-/postoperative patient’s condition in post-AMI PMR clinical scenarios.^2^^,^^17^^,^^18^ From our analysis, concomitant application of MCS has shown promising results, acting as a bridge to surgery or post-cardiotomy support. While IABP is still considered the first line of MCS in current ESC guidelines,^19^ application of V-A ECLS has been recently proposed and described in literature in post-AMI PMR patients as a potential tool to manage clinical conditions and provide benefit in terms of in-hospital survival.^2^

### Outcomes

In literature, the in-hospital mortality after post-AMI PMR surgery ranges between 20 and 40%.^7^^,^^9^^,^^10^^,^^11^ From the meta-analysis of Pala et al,^6^ the in-hospital mortality was higher in post-AMI PMR patients who underwent MVR than MVr surgery. No statistical differences were shown in our study about this. Anyway, post-AMI PMR patients underwent MVR, experienced a 5% higher mortality than MVr surgery. No publication in literature compared long-term survival between MVR and MVr in post-AMI PMR patients. In our study, there was no statistical significant difference in terms of long-term post-discharge outcome. More investigation and further clinical research are required to provide additional insight into the follow-up of patients who underwent surgery in post-AMI PMR. Despite this, it is our opinion that when feasible, the MVr surgery should be considered in post-AMI PMR, to avoid the possible prosthesis-related risks that could occur and impact over time after MVR surgery.^5^^,^^7^^,^^20^^,^^21^ The comparison of outcomes across these groups is meant to serve an illustrative purpose, emphasizing possible variations in patient characteristics and clinical courses, rather than implying any speculative claims about the superiority or preference of one surgical method over another. Our goal is not to recommend a specific treatment option, but to offer a descriptive summary of the population traits and their corresponding outcomes.

### Study limitation

Despite the benefits of our data from the CAUTION study, there are some limitations with the present study. First and foremost, the retrospective study nature carried biases such as the selection bias given by the observational nature. Second, the multicentre design required a data collection form with a limited number of variables to avoid missing data; thus, the possibility that non-reported variables could have influenced the results of the analysis cannot be completely ruled out.

For example, the database has no information on: the extent of the AMI, the degree of ventricular dysfunction, the operation performed, the extent of the coronary artery disease and the completeness of revascularization and durability of the MVr. Also, data concerning functional status as well as regular imaging modalities during the follow-up still missing. Third, the relatively small sample size underpowered the statistical analysis and could have limited the number of statistically significant variables. Concerning MVR, no data about the preservation of the subvalvular apparatus were reported. Although reporting late mortality, no sufficient data could be retrieved about the causes of late deaths or re-hospitalizations for cardiac causes at follow-up. We evaluated the effect of concomitant CABG on survival; however, we were unable to distinguish the target of revascularization, culprit, or non-culprit vessel. The CAUTION study enrolled only post-AMI PMR patients who underwent surgical treatment for post-AMI MCs; no information is available regarding excluded patients who were treated percutaneously or managed conservatively. Finally, although the present study collected a rarely large number of patients affected by such a rare complication as PMR, the sample size does not allow for reaching an adequate power to achieve clinically relevant differences, albeit providing some interesting and suggestive hints about this peculiar population. Larger and randomized multicentre prospective studies are required to identify independent predictors of mortality more accurately and to better clarify long-term survival; however, such studies are challenging to conduct and would probably face major ethical issues.

## CONCLUSION

According to the literature, in this study, MVR is the most performed procedure in post-AMI PMR. Male patients, haemodynamic instability, and preoperative IABP implant seem to be associated more frequently with MVR, while with MVr. MVr is more commonly performed in case of partial PMR, while complete PMR is usually treated with MVR. Concomitant CABG during mitral valve surgery for post-AMI PMR appears protective against in-hospital mortality, and therefore, additional surgical revascularization should be considered whenever possible in this group of patients. No significant differences in mortality were noticed during the follow-up. From the multivariable logistic regression model, no independent factors were associated with overall mortality.

## Supplementary Material

ezaf284_Supplementary_Data

## DATA AVAIBILITY

All relevant data are within the manuscript and its Supporting Information files.
