# Supplementary material for: Repair Versus Replacement in Mitral Valve Papillary Muscle Rupture: A Multicenter Study
Source: Eur J Cardiothorac Surg. 2025 Aug 22;67(9):ezaf284. doi: 10.1093/ejcts/ezaf284 (PMC12417086; doi:10.1093/ejcts/ezaf284)
Supplement: ezaf284_Supplementary_Data [file ezaf284_supplementary_data.docx]

**Supplementary Material**

**Figure S1: Inclusion criteria CAUTION STUDY**

AMI: acute myocardial infarction; PMR: papillary muscle rupture

**Figure S2**

**
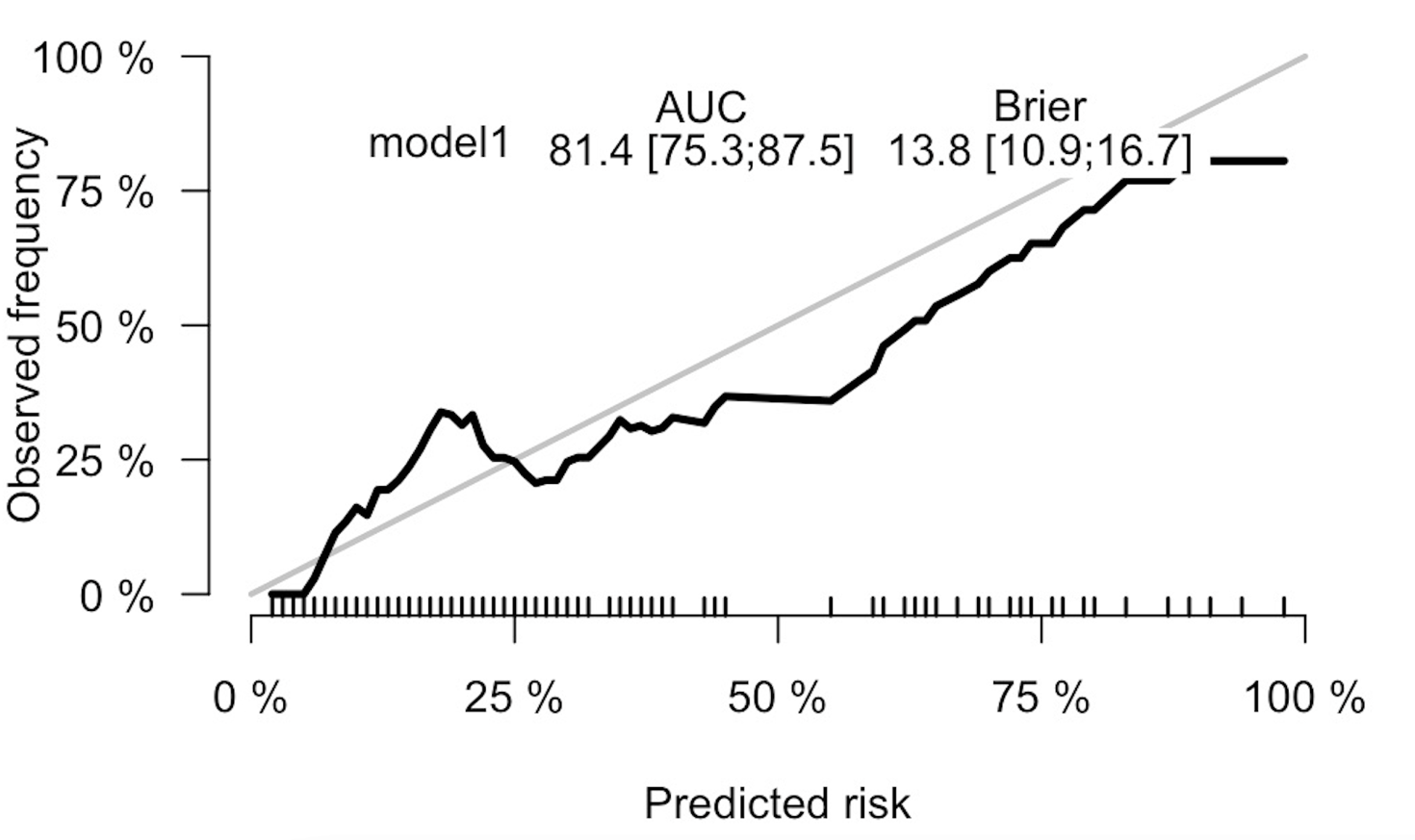
**

**Table 1S: In-hospital mortality predictors for post-acute myocardial infarction papillary muscle rupture**

|  | OR | 95% C.I. | | p-value |
| --- | --- | --- | --- | --- |
| Age | 1.044 | 1.008 | 1.08 | 0.015 |
| Male | 1.21 | 0.574 | 2.55 | 0.616 |
| LVEF (%) | 0.978 | 0.95 | 1.006 | 0.12 |
| Instability | 0.515 | 0.187 | 1.415 | 0.198 |
| Cardiac tamponade | 1.06 | 0.207 | 5.421 | 0.944 |
| IABP | 0.928 | 0.403 | 2.138 | 0.86 |
| ECMO | 2.27 | 0.521 | 9.894 | 0.275 |
| Previous thrombolysis | 2.372 | 0.445 | 12.642 | 0.312 |
| PMR complete | 0.638 | 0.322 | 1.263 | 0.197 |
| MVr | 0.651 | 0.25 | 1.695 | 0.379 |
